# Supplementary material for: From sequence to enzyme mechanism using multi-label machine learning
Source: BMC Bioinformatics. 2014 May 19;15:150. doi: 10.1186/1471-2105-15-150 (PMC4229970; doi:10.1186/1471-2105-15-150)
Supplement: Additional file 2 — Java code of ml2db. Additional file ml2db_code.tar.gz contains the Java source code to run the multi-label machine learning experiments and save the results to database. The code’s Javadoc is included. [file 1471-2105-15-150-S2.zip › additional file 2/ml2db/utils/doc/uk/ac/standrews/utils/main/database/TableWriter.html]

TableWriter


JavaScript is disabled on your browser.


- Overview
- Package
- Class
- Use
- Tree
- Deprecated
- Index
- Help

- Prev Class
- Next Class

- Frames
- No Frames

- All Classes

- Summary:
- Nested |
- Field |
- Constr |
- Method

- Detail:
- Field |
- Constr |
- Method


uk.ac.standrews.utils.main.database

## Class TableWriter

- java.lang.Object
- - uk.ac.standrews.utils.main.database.Manager
  - - uk.ac.standrews.utils.main.database.DbManager
    - - uk.ac.standrews.utils.main.database.TableManager
      - - uk.ac.standrews.utils.main.database.TableWriter

- All Implemented Interfaces:
  :   java.io.Serializable

  ---

    

  ```
  public class TableWriter
  extends TableManager
  ```

  A TableWriter object contains all the information to create, fill and delete
  a database table.

  Version:
  :   24-Mar-2007
      TODO create hierarchy?: table > table from file

  Author:
  :   Luna De Ferrari

  See Also:
  :   Serialized Form

- - ### Field Summary

    - ### Fields inherited from class uk.ac.standrews.utils.main.database.DbManager

      `m_dbWriter`
  - ### Constructor Summary

    Constructors

    Constructor and Description

    `TableWriter(Table table)`

    Constructor 2: takes the table name and a list of fields
  - ### Method Summary

    Methods

    | Modifier and Type | Method and Description |
    | --- | --- |
    | `java.lang.String` | `getInsertSql(TableRow row)` Returns the sql "insert into" statement for the row |
    | `java.lang.String` | `getLoadDataInfileSql(java.lang.String fileName, java.lang.String fieldTerminator, java.lang.String lineTerminator, int numberOfInitialLinesToIgnore, java.lang.String commaSeparatedListOfColumns)` |
    | `java.lang.Integer` | `insertReturnsKey(TableRow row)` Executes an sql 'insert into' operation. |
    | `boolean` | `writeFileToTable(java.lang.String fileName, java.lang.String fieldTerminator, java.lang.String lineTerminator, int numberOfInitialLinesToIgnore, java.lang.String commaSeparatedListOfColumns)` Writes a data file to table using LOAD DATA LOCAL INFILE return true if the statement was executed |
    | `java.lang.Integer` | `writeRowToTable(TableRow row)` Insert the row values into the appropriate database table |

    - ### Methods inherited from class uk.ac.standrews.utils.main.database.TableManager

      `getTable, getTableName`
    - ### Methods inherited from class uk.ac.standrews.utils.main.database.DbManager

      `closeConnection, connectionIsValid, getDatabaseName, getDbConnection, getDbCreator, getDbReader, getDbWriter, refreshConnection, runAnalyzeTable`
    - ### Methods inherited from class java.lang.Object

      `equals, getClass, hashCode, notify, notifyAll, toString, wait, wait, wait`

- - ### Constructor Detail


    - #### TableWriter

      ```
      public TableWriter(Table table)
      ```

      Constructor 2: takes the table name and a list of fields

      Parameters:
      :   `tableManager` - the table manager
  - ### Method Detail


    - #### getInsertSql

      ```
      public java.lang.String getInsertSql(TableRow row)
      ```

      Returns the sql "insert into" statement for the row

      Parameters:
      :   `row` -

      Returns:
      :   the sql "insert into" statement for the row


    - #### getLoadDataInfileSql

      ```
      public java.lang.String getLoadDataInfileSql(java.lang.String fileName,
                                          java.lang.String fieldTerminator,
                                          java.lang.String lineTerminator,
                                          int numberOfInitialLinesToIgnore,
                                          java.lang.String commaSeparatedListOfColumns)
      ```


    - #### insertReturnsKey

      ```
      public java.lang.Integer insertReturnsKey(TableRow row)
      ```

      Executes an sql 'insert into' operation. And returns the auto-generated
      key for the inserted row.

      Parameters:
      :   `row` - a table row (columns + values)

      Returns:
      :   the auto-generated key for the inserted row.


    - #### writeFileToTable

      ```
      public boolean writeFileToTable(java.lang.String fileName,
                             java.lang.String fieldTerminator,
                             java.lang.String lineTerminator,
                             int numberOfInitialLinesToIgnore,
                             java.lang.String commaSeparatedListOfColumns)
      ```

      Writes a data file to table using LOAD DATA LOCAL INFILE return true if
      the statement was executed


    - #### writeRowToTable

      ```
      public java.lang.Integer writeRowToTable(TableRow row)
      ```

      Insert the row values into the appropriate database table

      Returns:
      :   the table auto-generated key for the inserted row (or null if the
          row was not inserted)


- Overview
- Package
- Class
- Use
- Tree
- Deprecated
- Index
- Help

- Prev Class
- Next Class

- Frames
- No Frames

- All Classes

- Summary:
- Nested |
- Field |
- Constr |
- Method

- Detail:
- Field |
- Constr |
- Method
